# High background in ELISpot assays is associated with elevated levels of immune activation in HIV‐1‐seronegative individuals in Nairobi

**DOI:** 10.1002/iid3.231

**Published:** 2018-07-04

**Authors:** Amy Y. Liu, Stephen C. De Rosa, Brandon L. Guthrie, Robert Y. Choi, Rose Kerubo‐Bosire, Barbra A. Richardson, James Kiarie, Carey Farquhar, Barbara Lohman‐Payne

**Affiliations:** ^1^ Department of Epidemiology University of Washington Seattle Washington USA; ^2^ Department of Laboratory Medicine University of Washington Seattle Washington USA; ^3^ Vaccine and Infectious Disease Division Fred Hutchinson Cancer Research Center Seattle Washington USA; ^4^ Department of Medicine University of Washington Seattle Washington USA; ^5^ Centre for Public Health Research Kenya Medical Research Institute Nairobi Kenya; ^6^ Department of Global Health University of Washington Seattle Washington USA; ^7^ Department of Biostatistics University of Washington Seattle Washington USA; ^8^ Department of Obstetrics and Gynaecology Kenyatta National Hospital Nairobi Kenya; ^9^ Department of Paediatrics and Child Health University of Nairobi Nairobi Kenya

**Keywords:** Flow cytometry, HIV‐seronegative, IGRA

## Abstract

**Introduction:**

Spontaneous interferon‐γ (IFNγ) released detected by enzyme‐linked immunospot (ELISpot) assays may be a biological phenomenon. Markers of immune activation levels were assessed as correlates of high background among individuals in Kenya.

**Methods:**

Couples concordantly seronegative for HIV‐1 were enrolled. IFN‐γ ELISpot assays were conducted and negative control wells were categorized as having either high or low background (≥50 and <50 SFU/10^6^ peripheral blood mononuclear cells [PBMC], respectively). PBMC were stained for CD4, CD8, and immune activation markers (CD38 and HLA‐DR) and analyzed using flow cytometry. Proportions of activated T‐cells were compared between those with low and high background by Mann–Whitney U test. Correlates of background SFU and immune activation were assessed using regression models.

**Results:**

Among 58 individuals, 14 (24%) had high background. Frequencies of CD4^+^CD38^+^HLA‐DR^+^ and CD8^+^CD38^+^HLA‐DR^+^ cells were higher in individuals with high background compared to those with low background (*P *= 0.02). Higher background SFU was associated with history of sexually transmitted infections (*P *= 0.03), and illness in the past 3 months (*P *= 0.005), in addition to increased levels of activated CD4^+^ and CD8^+^ cells (*P* range = 0.008–0.03). Female gender and male circumcision decreased levels of CD4^+^ and CD8^+^ immune activation (*P* range = 0.002–0.03). Additionally, higher background SFU and activated CD4^+^ and CD8^+^ cells were individually associated with positive ELISpot responses to HIV‐1 peptide pools (*P* range = 0.01–0.03).

**Conclusions:**

These findings suggest that increased basal immune responses may be a biological mechanism contributing to higher background ELISpot SFU. Systematic exclusion of data from individuals with increased background in IFN‐γ release assays may bias results in population‐based studies.

## Introduction

The IFN‐γ enzyme‐linked immunospot (ELISpot) assay is widely used to quantify viral, tumor, allo‐, and auto‐antigenic cellular immune responses in clinical and vaccine trials. This assay is quick, cost‐effective, and one of the most sensitive methods to detect antigen‐specific T cell responses 1, 2. Quality of ELISpot assay results depends on accurate detection of spot forming units (SFU) through good staining that allows discrimination of specific spots (signal) from non‐specific background spots (noise). Therefore, it is necessary to minimize background SFU in negative control wells and to maximize antigen‐induced spots in experimental wells to optimize the signal‐to‐noise ratio.

Methodological issues are commonly cited as possible reasons for spot production in negative control wells 3. Although the general consensus is that high SFU in unstimulated wells are due to assay‐specific problems and should be excluded from analyses, ELISpot assays are not standardized and exclusion criteria based on high background varies depending on protocols 3, 4, 5. However, biological factors may also influence basal IFN‐γ production detected ex vivo. Our group has repeatedly observed dramatic differences in negative control IFN‐γ responses from PBMC obtained from two individuals tested on one ELISpot plate, where the only variation in assay protocol occurred at the level of the sample. This observation propelled us to examine correlates of spontaneous IFN‐γ secretion in greater detail.

Studies have observed elevated immune activation among populations from developing countries compared to North American or European cohorts and environmentally driven factors, such as chronic infections, limited nutrition, and poor hygienic conditions, have been proposed as possible explanations for these findings 6, 7, 8, 9, 10, 11. We hypothesized that there may be a biological phenomenon mediated by increased immune activation that results in increased IFN‐γ secretion ex vivo, read out as higher SFU (background) in negative controls wells of ELISpot assays. We conducted a cross‐sectional study to assess the prevalence of high background among HIV‐1‐seronegative couples and to identify potentially modifiable biological correlates driving these background responses.

## Materials and Methods

### Study population

Couples in which both partners tested HIV‐1‐seronegative were recruited from voluntary counseling and testing centers in Nairobi, Kenya from 2007 to 2009. Enrolled couples reported having sex with their partner ≥3 times in the 3 months prior to screening and did not report any outside sexual partnerships, and women participants were not pregnant. Written informed consent was obtained from all study participants, and ethical approval was received from the Institutional Review Boards at the University of Washington and Kenyatta National Hospital. Couples were seen once in clinic at enrollment. During this visit, clinical staff collected blood and genital specimens and administered questionnaires to collect sociodemographic, sexual behavior, and self reported medical history data.

### Laboratory procedures

Couples were determined to be concordantly HIV‐1‐seronegative using the Determine HIV‐1/2 rapid test (Abbott, Toyko, Japan) and the Bioline HIV 1/2 rapid test (Standard Diagnostics, Gyeonggi‐do, Korea). HSV‐2 serology was determined using the HerpeSelect IgG ELISA kit (Focus Technologies, Cypress, CA, USA). Equivocal HSV‐2 test results were analyzed as either a negative or a positive result. Syphilis was tested using a rapid plasma reagin (RPR) test (Becton Dickinson [BD], San Jose, CA, USA); reactive tests were confirmed by *Treponema pallidum* haemagglutination assay (Randox, Crumlin, UK). For female participants the following tests were conducted: urine pregnancy tests (Quick Vue One Step hCG Urine Pregnancy kit, Quidel Corporation, San Diego, CA, USA), *Trichomonas vaginalis* cultures (In‐Pouch TV, Biomed Diagnostics, White City, OR, USA), and bacterial vaginosis (BV).

### IFN‐γ ELISpot assays

Blood samples from the couples were collected on the same day and batch processed to reduce introduction of within‐couple variability. ELISpot assays were conducted to evaluate the frequency of background and antigen stimulated SFU with a previously described protocol using Millipore plates (Millipore, Burlington, MA, USA) and Mabtech (Nacka Strand, Sweden) reagents 12. One × 10^5^ freshly isolated peripheral blood mononuclear cells (PBMC)/well were stimulated with phytohemagglutinin (PHA) (Murex Biotech Ltd., Dartford, UK) in triplicate as positive control wells, media alone in nine negative control wells, or HIV‐1 peptide pools in triplicate experimental wells. Twenty‐two peptide pools of 15‐mers overlapping by 10 amino acids spanning the HIV‐1 genome were derived from the HIV‐1 subtype A consensus sequence (Sigma‐Genosys, Burlington, MA, USA). Plates were read on a CTL ImmunoSpot S4 Core Analyzer, and analyzed using ImmunoSpot Software (Cellular Technology Ltd., Shaker Heights, OH, USA).

The background response, defined as the mean SFU in the nine negative control wells, was categorized as low (<50 SFU/10^6^ PBMC) or high (≥50 SFU/10^6^ PBMC). Background responses were examined both as a dichotomous (pre‐defined cutoffs above) and continuous (magnitude of responses) variable. HIV‐1‐stimulated SFU were defined as the average number of spots in triplicate wells minus background. IFN‐γ ELISpot responses were considered positive if experimental wells had ≥50 HIV‐1‐stimulated SFU/10^6^ PBMC and more than twice the background response. Individuals were defined as positive ELISpot responders if they had ≥1 peptide pool with a positive response.

### Immune activation assays

Immune activation markers were measured on fresh whole blood specimens. Specimens were stained with the following pre‐mixed four‐color fluorochrome‐conjugated antibody combinations: anti‐CD4‐FITC, anti‐CD38‐PE, anti‐CD3‐PerCP, anti‐HLA‐DR‐APC and anti‐CD8‐FITC, anti‐CD38‐PE, anti‐CD3‐PerCP, anti‐HLA‐DR‐APC (BD). Specimens were run on a four‐color FACSCalibur flow cytometer (BD), and flow cytometry data were analyzed and quality controlled using FlowJo software (Treestar, Ashland, OR, USA). Gates were set conservatively to capture high‐level expression of CD38/HLADR. Percentages of activated cells, defined as CD38^+^HLA‐DR^+^, CD38^‐^HLA‐DR^+^, or HLA‐DR^+^ (CD38^+^ or CD38^−^), in both CD4^+^ and CD8^+^ subsets were used in analyses.

### Statistical methods

Activated cells were compared between individuals with high and low background responses and between positive and negative ELISpot responders using Mann–Whitney U tests. Linear regression with robust standard errors was used to assess correlates of background response. Data were log_10_ transformed when examining magnitude of background SFU as a continuous variable to achieve a normal distribution. A set of characteristics was selected a priori as potential correlates of immune activation. Both partners of the couple reported number of sex acts with their study partner in the past month, and the mean number of acts reported by the couple was used for analysis. Also, any individual who self‐reported having a fever, diarrhea, vomiting, or a cough in the past 3 months was considered to have a recent illness. To determine correlates of immune activation, immune activation data were modeled as a proportion, and multivariate analyses were performed for each characteristic adjusting for gender and age using generalized linear models with logit link and robust standard errors. All analyses were conducted using Stata version 11.2 statistical software (College Station, TX, USA).

## Results

### Study population characteristics

Fifty‐eight individuals in monogamous, concordant HIV‐1‐sero*negative* relationships were included in the analyses. Among these individuals, 29 (50%) were females and the median age for all participants was 27 (interquartile range [IQR] 23–31) (Table 1). Median length of cohabitation was 1 year (IQR 0–7). Participants reported a median of four lifetime sexual partners (IQR 3–6) and five sex acts (IQR 3–12) with their study partner in the past month. From self‐reporting, 43 (74%) had unprotected sex in the past month, 12 (21%) had a history of sexually transmitted infections (STI), and 5 (9%) had an illness (fever, diarrhea, vomiting, or cough) in the past 3 months. Twenty (35%) individuals were HSV‐2 seropositive or had an equivocal result, and 24 (83%) of the men were circumcised. Among the women, 20 (69%) used birth control, the most common form was oral, injectable, or implanted hormonal contraceptives.

**Table 1:** Cohort characteristics of concordant HIV‐1‐seronegative couples

Characteristic	Median (IQR) or *n* (%) (*N* = 58)*
Female	29 (50)
Age	27 (23–31)
Years living together	1 (0–7)
Lifetime sexual partners	4 (3–6)
Sex acts^a^	5 (3–12)
Any unprotected sex^a^	43 (74)
History of STI	12 (21)
HSV‐2 serostatus	
Negative	37 (65)
Equivocal	5 (9)
Positive	15 (26)
Bacterial vaginosis	5 (31)
Recent illness^b^	5 (9)
Male circumcision	24 (83)
Birth control use	20 (69)
Hormonal birth control use^c^	13 (45)

IQR, interquartile range; STI, sexually transmitted infection.

* *N* = 29 for characteristics unique to men or women. Bacterial vaginosis results were available for 16 women.

^a^ With study partner in the past month.

^b^ Reported fever, diarrhea, vomiting, or cough in the past 3 months.

^c^ Hormonal birth control use defined as oral, injectable, or implant contraceptives.

### ELISpot background response and immune activation

**Figure 1:**
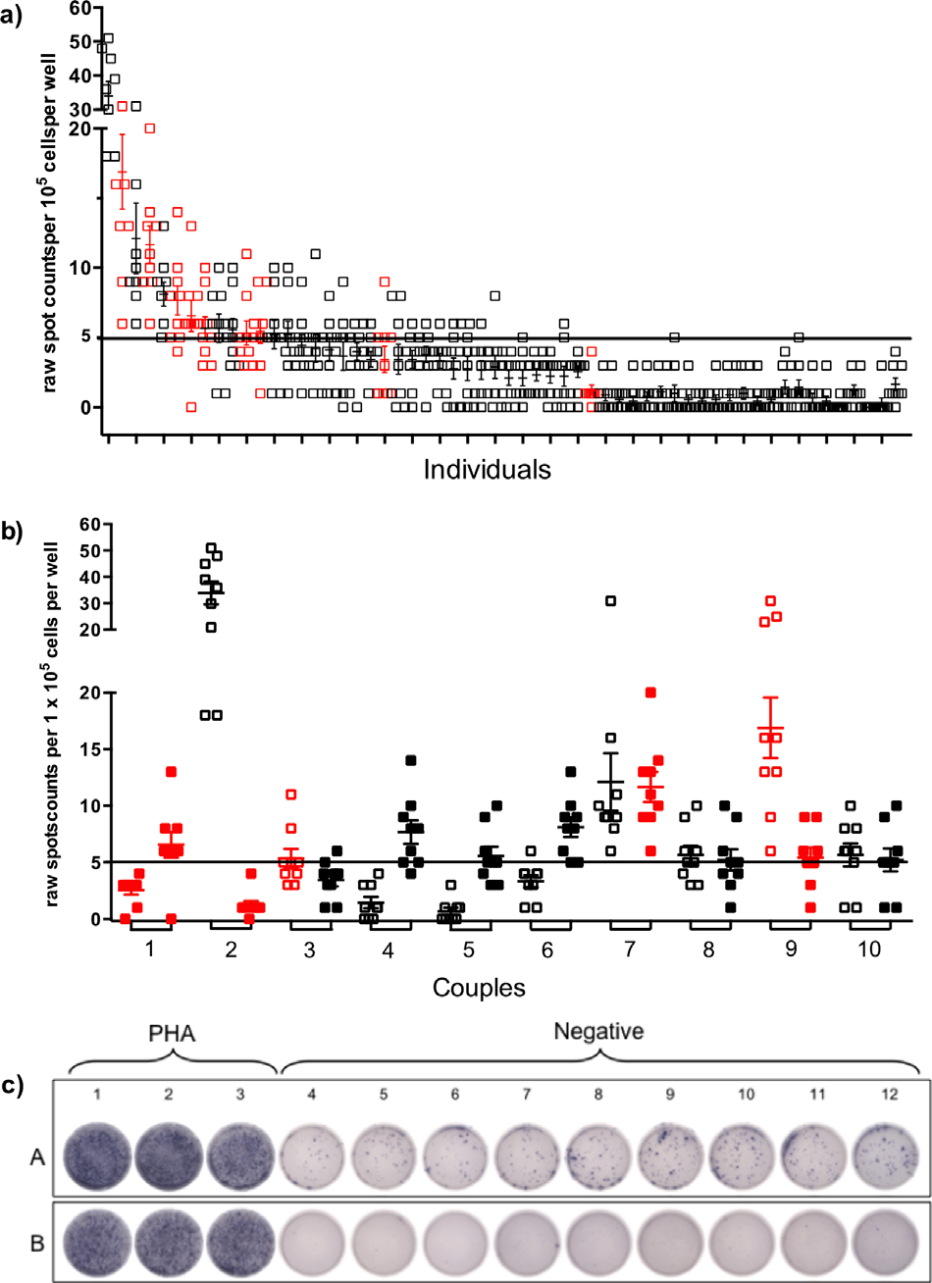
Background response of ELISpot assays among HIV‐1‐uninfected individuals. Negative control wells (replicates of nine) containing 10^5^ PBMC and media only were cultured overnight for determination of background IFN‐γ response. A reference line at five spots represents the cut‐off number for categorizing a high (≥5 SFU/10^5^ PBMC) versus low (<5 SFU/10^5^ PBMC) response. Individual wells (squares) and mean ± SEM are shown per person. Individuals scoring positive for HIV peptide responses (>2 × background and ≥ 50 spots) are indicated in red. a) Range of IFN‐γ responses from 58 HIV‐1‐seronegative individuals. b) Couples in which one or both individuals scored as high background by IFN‐γ response. c) ELISpot plate images from positive (columns 1–3) and negative control wells (columns 4–12) are shown for partners (A and B) of a couple run with the same assay conditions. Partner A was categorized as having a high background response and partner B as a low background response.

Of the 58 low‐risk participants, the median magnitude of background response was 24 SFU/10^6^ PBMC (IQR 10–44). Replicate spot counts per well based on input cell number of 1 × 10^5^ cells are shown in Figure 1a, demonstrating the range of individual variability within the cohort. When ELISpot background responses were dichotomously categorized, the majority (*n* = 44,76%) individuals had low background secretion of INF*‐*γ, however, 14 (24%) individuals were categorized as having high background with a median of 61 SFU/10^6^ PBMC (IQR 54–121), shown in detail in Figure 1b as partners within each couple. Approximately half the individuals were in partnerships with other “high background” individuals, while the other half were in partnerships with individuals characterized as low background on the same plate, as shown in Figure 1c.

**Figure 2:**
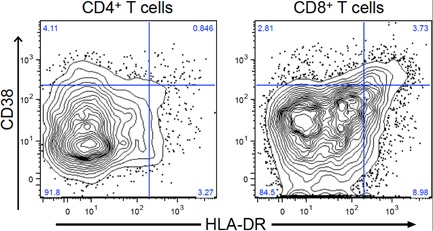
Example of staining profile for immune activation markers CD38^+^ and HLA‐DR^+^ on CD4^+^ and CD8+ T cells. CD38^+^ and HLA‐DR^+^ staining among CD4^+^ and CD8+ T cells for an individual. Quadrants were placed conservatively high as activated cells generally have bright expression of these markers and this placement achieves a restrictive estimate of activated cells, reducing the likelihood of falsely categorizing cells as activated.

**Figure 3:**
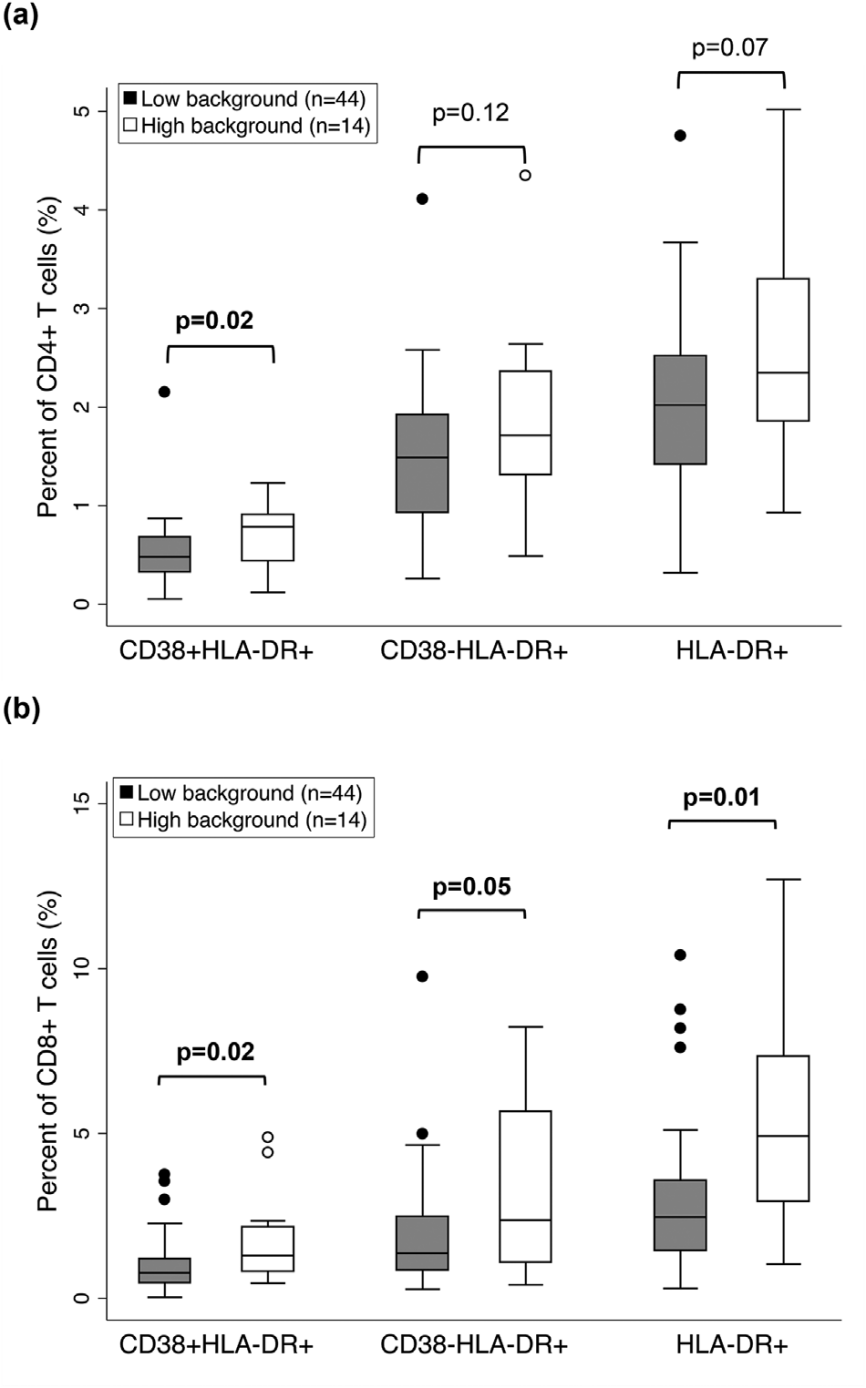
Immune activation markers by ELISpot background response. Frequency of activated cells in (a) CD4^+^ T and (b) CD8^+^ T cell subsets are shown for individuals with low (gray box plot, <50 SFU/10^6^ PBMC) and high (open box plot, ≥50 SFU/10^6^ PBMC) background response. Upper, middle, and lower lines of the box show group 75th percentile, median, and 25th percentile, respectively, individual outliers indicated by solid circles. *P*‐values compare the distribution of the percent of activated cells for individuals with low background to those with high background, based on the Mann–Whitney U test.

T cells were surface stained with immune activation markers CD38^+^ and HLA‐DR^+^ and analyzed to determine the level of immune activation. Depending on differential CD38^+^ and HLA‐DR^+^ phenotypes, 10–40% more CD8^+^ T cells expressed markers of activation compared to CD4+ T cells (Fig. 2). When compared by ELISpot background response, frequency of CD4^+^ T cells co‐expressing CD38^+^ and HLA‐DR^+^ was significantly elevated in individuals with high background compared to those with low background (*P* = 0.02) (Fig. 3a). Similarly, compared to individuals with low background, those with high background had higher frequencies of CD8^+^ HLA DR^+^ T cells with and without CD38 expression (*P* = 0.02 and *P* = 0.05, respectively) (Fig. 3b).

### Correlates of IFN‐γ ELISpot background response

Univariate and multivariate models were used to evaluate the association between ELISpot background, immune activation and sociodemographic variables. ELISpot background was modeled as a continuous variable to increase the power of detecting an association and because a biologically meaningful cutoff is not known. Variables linked to infectious processes were associated with increased background. In univariate models, individuals who reported a history of STI or recent illness had higher magnitudes of log_10_ background SFU compared to those who did not (*β* = 0.82, 95% confidence interval [CI] = 0.07–1.56, *P* = 0.03; *β* = 0.98, 95% CI = 0.31–1.65, *P* = 0.005, respectively) (Table 2). There was also a trend toward higher log_10_ background SFU for individuals who were HSV‐2 positive or equivocal (*P* = 0.07).

**Table 2:** Correlates of magnitude of ELISpot background responses

Characteristic (*N* = 58)*	Coeff	95% CI	*P*‐value
Univariate analyses^a^			
Female	−0.03	−0.68 to 0.61	0.92
Age (per 5 years)	0.03	−0.14 to 0.20	0.73
Years living together	−0.03	−0.18 to 0.11	0.64
High sexual partners^b^	0.22	−0.42 to 0.87	0.49
Sex acts^c^	−0.001	−0.05 to 0.05	0.97
Any unprotected sex^c^	0.54	−0.19 to 1.27	0.15
History of STI	0.82	0.07 to 1.56	**0.03**
HSV‐2 seropositive (positive or equivocal)^d^	0.60	−0.05 to 1.25	0.07
Bacterial vaginosis	−0.31	−1.45 to 0.83	0.57
Recent illness^e^	0.98	0.31 to 1.65	**0.005**
Male circumcision	−0.35	−1.27 to 0.56	0.43
Hormonal birth control use^f^	−0.58	−1.51 to 0.36	0.22
CD4^+^CD38^+^HLA‐DR^+^	0.76	−0.44 to 1.96	0.21
CD4^+^CD38^−^HLA‐DR^+^	0.42	0.06 to 0.79	**0.03**
CD4^+^HLA‐DR^+^	0.39	0.08 to 0.71	**0.02**
CD8^+^CD38^+^HLA‐DR^+^	0.23	−0.05 to 0.51	0.10
CD8^+^CD38^−^HLA‐DR^+^	0.16	0.04 to 0.28	**0.008**
CD8^+^HLA‐DR^+^	0.13	0.03 to 0.23	**0.01**
Multivariate analyses^g^			
History of STI	0.70	0.07 to 1.33	**0.03**
Recent illness	0.95	0.51 to 1.39	**<0.001**
CD4^+^CD38^+^HLA‐DR^+^	0.82	−0.28 to 1.92	0.14
Recent illness	0.92	0.37 to 1.46	**0.001**
CD4^+^CD38^−^HLA‐DR^+^	0.40	0.05 to 0.75	**0.03**
Recent illness	0.96	0.45 to 1.48	**<0.001**
CD4^+^HLA‐DR^+^	0.39	0.08 to 0.69	**0.01**
History of STI	0.73	0.06 to 1.40	**0.03**
Recent illness	0.95	0.48 to 1.42	**<0.001**
CD8^+^CD38^+^HLA‐DR^+^	0.27	0.00 to 0.53	**0.05**
History of STI	0.67	0.02 to 1.32	**0.04**
Recent illness	0.91	0.48 to 1.34	**<0.001**
CD8^+^CD38^−^HLA‐DR^+^	0.17	0.05 to 0.28	**0.004**
History of STI	0.68	0.05 to 1.32	**0.04**
Recent illness	0.95	0.52 to 1.39	**<0.001**
CD8^+^HLA‐DR^+^	0.13	0.04 to 0.23	**0.005**

Coeff, beta coefficient from regression model; CI, confidence interval; STI, sexually transmitted infections. **BOLD** indicates *P* ≤ 0.05.

* *N* = 29 for characteristics unique to men or women. Bacterial vaginosis results were available for 16 women.

^a^ Association between log_10_ background SFU and each covariate is examined separately in unadjusted models.

^b^ Lifetime sexual partners dichotomized at median: <4 and ≥4.

^c^ With study partner in the past month.

^d^ HSV‐2 seropositive defined as positive or equivocal HSV‐2 test results.

^e^ Reported fever, diarrhea, vomiting, or cough in the past 3 months.

^f^ Hormonal birth control use defined as oral contraceptives, injectables, or implants.

^g^ Multivariate analyses included history of STI, recent illness, and immune activation as covariates; models with CD4^+^C38^−^HLA‐DR^+^ and CD4^+^HLA‐DR^+^ cells only included recent illness due to co‐linearity with history of STI.

Furthermore, T cell activation was associated with higher magnitude of background response, similar to the previous associations when background response was examined as a dichotomous variable. Among CD4^+^ T cells, an increase in proportions of CD38^‐^HLA‐DR^+^ and total HLA‐DR^+^ cells were associated with an increase in log_10_ background SFU (*β* = 0.42, 95% CI = 0.06–0.79, *P* = 0.03; *β* = 0.39, 95% CI = 0.08–0.71, *P* = 0.02, respectively). Analogous associations were found with higher CD8^+^ T cell activation (CD38^‐^HLA‐DR^+^: *β* = 0.16, 95% CI = 0.04–0.28, *P* = 0.008; HLA‐DR^+^: *β* = 0.13, 95% CI = 0.03–0.23, *P* = 0.01). To determine whether immune activation or infectious processes was driving the increased background responses, multivariate analyses were conducted. Infectious processes and immune activation both remained statistically significant correlates of magnitude of background SFU (range of *P*‐values: <0.001–0.05), except for the model including CD4^+^CD38^+^HLA‐DR^+^ cells.

### Correlates of immune activation

Having seen an association between T cell activation and IFN‐γ ELISpot background response, additional analyses were conducted to examine correlates of immune activation. These analyses were adjusted for gender and age. Females were more likely to have activated CD4^+^CD38^−^HLA‐DR^+^ (aOR = 0.73, 95% CI = 0.58–0.91, *P* = 0.006) and total HLA‐DR^+^ (aOR = 0.68, 95% CI = 0.52–0.87, *P* = 0.002) compared to males (Table 3). Additionally, trends were observed indicating that women who reported any unprotected sex in the past month were more likely to have activated CD38^+^HLA‐DR^+^ cells (*P* = 0.10), and those who had BV were more likely to have a higher proportion of cells expressing HLA‐DR^+^ (*P* = 0.09).

**Table 3:** Correlates of immune activation

Correlate^a^ (*N *= 58)*	aOR	95% CI	*P*‐value	aOR	95% CI	*P*‐value	aOR	95% CI	*P*‐value
	CD4^+^CD38^+^HLA‐DR^+^			CD4^+^CD38^−^HLA‐DR^+^			CD4^+^HLA‐DR^+^		
Female^b^	0.88	0.68–1.13	0.31	0.73	0.58–0.91	**0.006**	**0.68**	**0.52–0.87**	**0.002**
Years living together	1.06	0.87–1.28	0.58	1.00	0.90–1.11	0.95	0.98	0.88–1.08	0.67
High sexual partners^c^	1.03	0.65–1.64	0.90	1.11	0.89–1.38	0.37	1.13	0.94–1.37	0.20
Sex acts^d^	0.99	0.97–1.01	0.38	1.00	0.98–1.01	0.83	1.00	0.98–1.02	0.93
Any unprotected sex^d^	1.31	0.95–1.81	0.10	1.16	0.91–1.49	0.24	1.11	0.85–1.46	0.44
History of STI	1.19	0.87–1.65	0.28	1.22	0.96–1.55	0.11	1.23	0.93–1.63	0.16
HSV‐2 seropositive (positive or equivocal)^e^	1.21	0.85–1.70	0.29	0.96	0.76–1.20	0.69	0.87	0.70–1.09	0.22
Bacterial vaginosis	0.91	0.40–2.04	0.82	1.30	0.73–2.33	0.37	1.57	0.93–2.65	0.09
Recent illness^f^	0.84	0.56–1.26	0.39	0.99	0.76–1.29	0.93	1.04	0.82–1.34	0.73
Male circumcision	0.82	0.60–1.12	0.21	1.04	0.78–1.37	0.81	1.13	0.79–1.60	0.50
Hormonal birth control use^g^	0.80	0.52–1.22	0.29	0.80	0.59–1.10	0.17	0.81	0.59–1.11	0.19

	CD8^+^CD38^+^HLA‐DR^+^			CD8^+^CD38^−^HLA‐DR^+^			CD8^+^HLA‐DR^+^		
Female^b^	1.02	0.70–1.49	0.90	0.78	0.49–1.25	0.31	0.86	0.58–1.27	0.44
Years living together	1.14	0.90–1.44	0.29	1.09	0.90–1.31	0.37	1.07	0.92–1.34	0.29
High sexual partners^c^	0.78	0.37–1.65	0.52	0.87	0.48–1.60	0.66	0.84	0.44–1.58	0.58
Sex acts^d^	0.98	0.95–1.01	0.30	0.99	0.96–1.02	0.67	0.99	0.96–1.02	0.50
Any unprotected sex^d^	1.11	0.69–1.78	0.67	1.32	0.69–2.52	0.40	1.25	0.73–2.13	0.42
History of STI	1.16	0.61–2.23	0.65	1.01	0.59–1.74	0.97	1.06	0.63–1.77	0.84
HSV‐2 seropositive (positive or equivocal)^e^	1.18	0.72–1.94	0.52	1.00	0.62–1.61	0.99	1.06	0.67–1.68	0.79
Bacterial vaginosis	1.34	0.75–2.42	0.32	2.16	0.96–4.86	0.06	1.78	0.95–3.33	0.07
Recent illness^f^	0.74	0.35–1.59	0.45	0.83	0.36–1.94	0.67	0.80	0.35–1.80	0.59
Male circumcision	**0.55**	**0.32–0.94**	**0.03**	1.11	0.67–1.85	0.69	0.87	0.53–1.41	0.56
Hormonal birth control use^g^	0.65	0.41–1.05	0.08	0.68	0.40–1.16	0.16	0.67	0.42–1.05	0.08

aOR, adjusted odds ratio; CI, confidence interval; STI, sexually transmitted infections. **BOLD** indicates *P* ≤ 0.05.

* *N* = 29 for characteristics unique to men or women. Bacterial vaginosis results were available for 16 women.

^a^ Association between frequency of immune activation marker and each covariate is examined separately in multivariate models adjusting for gender and age.

^b^ Adjusted for age.

^c^ Lifetime sexual partners dichotomized at median: <4 and ≥4.

^d^ With study partner in the past month.

^e^ HSV‐2 seropositive defined as positive or equivocal HSV‐2 test results.

^f^ Reported fever, diarrhea, vomiting, or cough in the past 3 months.

^g^ Hormonal birth control use defined as oral contraceptives, injectables, or implants.

A different set of characteristics was observed to be associated with CD8^+^ immune activation. Circumcised men were less likely to have activated CD38^+^HLA‐DR^+^ cells (aOR = 0.55, 95% CI = 0.32–0.94, *P* = 0.03). Furthermore, there were trends for females who were positive for BV to have increased proportions of total HLA‐DR^+^ cells (*P* = 0.07) and for those who reported hormonal contraceptive use to have decreased frequency of total HLA‐DR^+^ cells (*P* = 0.08).

### Background and immune activation by antigen‐stimulated IFN‐γ ELISpot response

**Figure 4:**
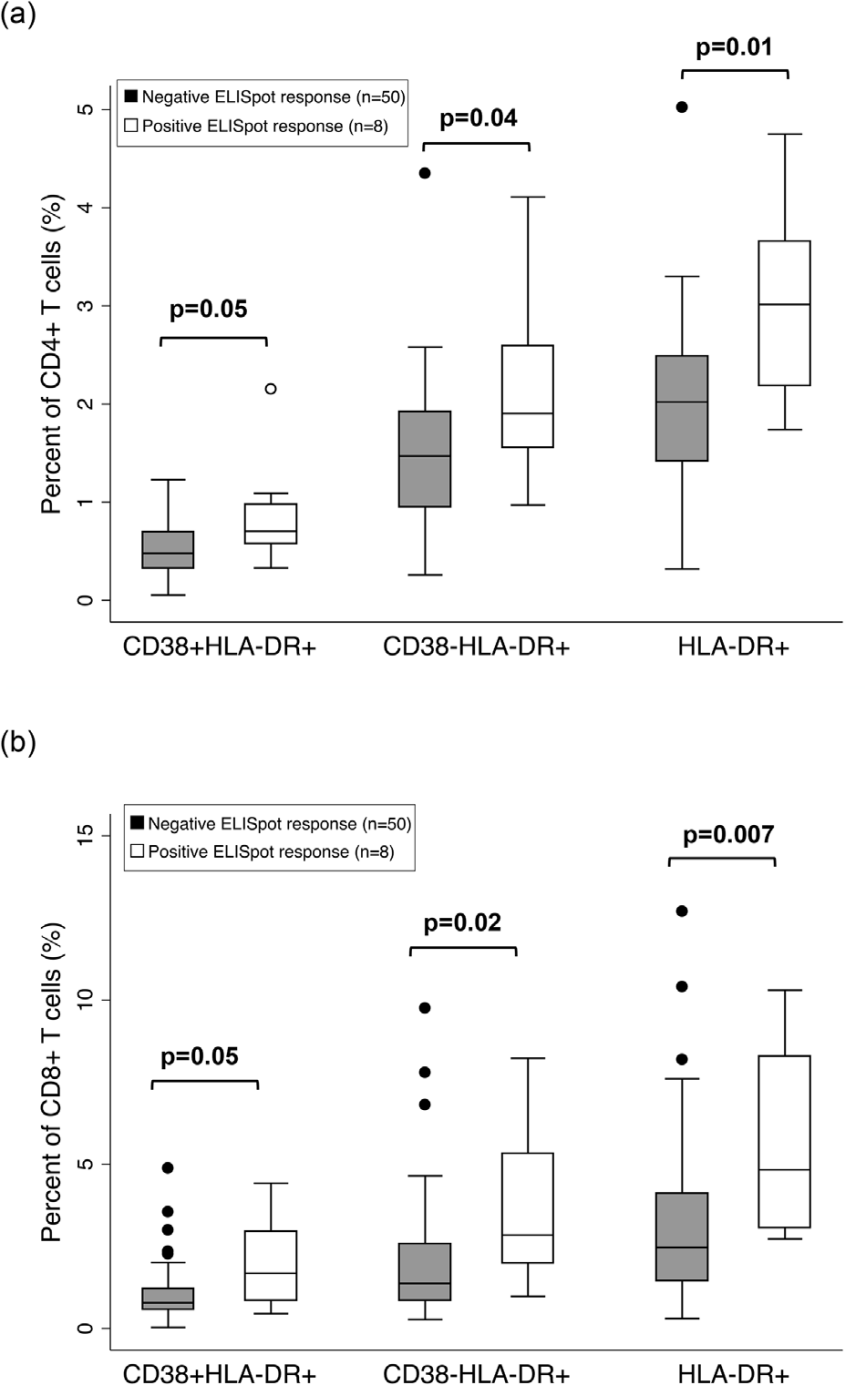
Immune activation markers by ELISpot response. Frequency of activated cells in (a) CD4^+^ T and (b) CD8^+^ T cell subsets are shown for individuals with negative (filled box) and positive (open box) ELISpot response, defined as twofold over background and at least 50 SFU/10^6^ cells. Upper, middle, and lower lines of the box show group 75th percentile, median, and 25th percentile, respectively. *P*‐values compare the distribution of the percent of activated cells for individuals with negative response to those with positive response, based on the Mann–Whitney U test.

To assess the association between immune activation and ELISpot response to antigen‐specific stimulation, levels of activated cells were compared between individuals who had positive HIV‐1 ELISpot results and those who had negative results. The majority (50/58, 86%) had negative IFN‐γ responses to HIV‐1 peptides and 9 of these 50 (18%) had high background. In comparison eight participants (14%) were categorized as positive responders and among those 63% (5/8) had a high background response. Thus, individuals with a high background response were ∼7 times more likely to have a positive IFN‐γ ELISpot response to HIV peptide pools (OR = 7.6, *P* = 0.01). Additionally, a strong positive correlation was seen between magnitude of background response and magnitude of HIV‐1‐stimulated SFU (Spearman's *ρ *= 0.43, *P* < 0.001, data not shown). Furthermore, CD38 + HLADR+ expression in both CD4^+^ and CD8^+^ T cell subsets were significantly elevated in individuals who had a positive ELISpot response compared to those who did not (Fig. 4). The association between presence of a positive response and magnitude of background response remained statistically significant (*P* ≤ 0.05) after adjusting for frequency of CD8 + HLADR+ activated cells, except when adjusting for the CD4^+^HLA‐DR^+^ phenotype, which showed a trend (*P* = 0.08, data not shown).

## Discussion

In this study we found of high spontaneous IFN‐γ release in ELISpot assays conducted on PBMC from ∼25% of individuals and these individuals were more likely to have elevated levels of immune activation in both CD4^+^ and CD8^+^ T cells. Naïve T cells differentiate into memory T cells after being activated 13, and the activated effector cells are then responsible for producing cytokines in vivo, most typically only during the acute immune response before the cells die or differentiate into resting memory cells. As other studies in developing countries have speculated, our participants are likely exposed to a wide spectrum of pathogens that may result in persistent infections that cause chronic immune activation. Thus, these findings support the hypothesis that highly activated T cells contribute to spontaneous IFN‐γ production ex vivo detected in IFN‐γ release assays.

We found that a history of having a STI or an illness in the past 3 months was significantly associated with high background SFU, even after adjusting for immune activation. We expected these markers of infection to lead to an increase in activated cells, and subsequently, elevated background response; however, the immune activation markers that were measured may not have captured the whole T cell activation pathway. In addition, since unfractionated PBMC were added to our ELISpot assays, the cells of the innate immune system, particularly NK cells, may also be responsible for IFN‐γ production detected in the negative control wells 14.

Interestingly, different correlates were found for high background response and immune activation. Women had lower median frequencies of activated CD4^+^CD38‐HLADR+ cell phenotypes compared to men, which is consistent with a previous study that found African American women had decreased levels of the immune activation marker neopterin compared to African American men 15. The preponderance of HLA‐DR+ cells relative to CD38+ populations was unexpected. Male circumcision was associated with decreased immune activation. Since results from large randomized clinical trials showed a strong association between male circumcision and protection against HIV‐1 infection 16, 17, 18, it is not surprising that circumcised men have lower levels of activated T cells. Furthermore, this finding may provide an explanation as to why uncircumcised men had a higher risk for HIV‐1 infection in the Step Study 19.

To better understand the detection of HIV‐1‐stimuated responses in our HIV‐1‐uninfected participants, we determined the effect of background SFU and immune activation on the ELISpot response. We observed higher proportions of activated T cells were associated with positive ELISpot responses. These results are similar to a previous study that showed HIV‐1‐negative Kenyan women who had elevated T cell immune activation expressed significantly increased IFN‐γ production when stimulated with superantigen staphylococcal enterotoxin B 11. Our results suggest that either immune activation leads to non‐specific IFN‐γ release that may interfere with the classification of having a positive response due to background (i.e., false positives), or immune activation leads to higher levels of cross‐reactivity to HIV‐1 peptides. Furthermore, we found that individuals with higher background SFU were more likely to have a higher magnitude HIV‐1‐stimulated response. When including both background response and CD8 + CD38^+^HLA‐DR^+^ immune activation in the model for predictors of a positive antigen stimulated ELISpot response, background remained the only statistically significant predictor. The results from adjusted models suggest activated T cells are hyper‐responsive, secreting higher levels of cytokines when stimulated, and thereby producing false positive ELISpot responses mediated through higher background responses. While these findings support the idea that activated cells may have contributed to false positive HIV‐1‐specific cellular responses through background response, it does not preclude the possibility of the participants being unwilling to acknowledge exposure to HIV‐1 from outside partnerships, exposure that can lead to HIV‐1 specific responses 20.

Our data suggest criteria for defining positive ELISpot responses balance the risk of false positive responses with the risk of introducing significant bias through the selectively removal of data from individuals with high background. Chronic immune activation can lead to immune dysfunctions such as anergy, activation‐induced cell death, cytokine dysregulation, and impaired signal transduction 8, 21, 22, 23, and previous studies have demonstrated the importance of the host's pre‐existing immune status in influencing the efficacy of immune responses to vaccine or pathogen challenge 24, 25. It has been proposed that the activation state of the participants may be a possible explanation for the unexpected increase in HIV‐1 acquisition found in the Step study 19, 26. Individuals who have elevated immune activation levels may have difficulty mounting an effective immune response, so excluding these individuals may significantly bias study findings, especially for vaccine trials.

While a strength of this study was performing ELISpot assays on fresh samples to optimize sensitivity, rerunning the assays on frozen aliquots may yield different findings. Due to limited numbers of PBMC, we were not able to compare responses between fresh and frozen samples. Another limitation of this study is the limited data on co‐infections within this population. Helminth infections, malaria, and TB are highly endemic in Kenya and cause a chronic immune activation state, but we were unable to diagnosis these conditions within this study. Furthermore, unfractionated PBMC were used in the assay, and together with the use of 15‐mers peptides overlapping by 10 amino acids, the cell type responsible for IFN‐γ secretion was not identified. While these factors may limit the scope of our conclusions, they do not affect the foundational findings.

In conclusion, our results suggest that elevated levels of immune activation, along with previous infections, are associated with both higher background IFN‐γ secretion and positive HIV‐1 stimulated ELISpot responses in a small but significant percent of the general population. Additionally, different correlates were found to contribute to T cell activation, highlighting the intricacies of the immune response. This study indicates that conditions that lead to a persistently activated immune state in HIV‐1‐seronegative individuals in Kenya can have a dramatic effect on immunological assays, and as such, the pre‐existing immune profile of populations should be considered when developing and testing assays for measuring immunogenicity of pathogens or potential vaccines.

## Ethical Statement

Written informed consent was obtained from all study participants. This study received ethical approval from the institutional review boards of the University of Washington and the University of Nairobi and was conducted according to the guidelines set forth by the United States Department of Health and Human Services.

## Authors’ Contributions

AYL conducted the assays and analyses and wrote the manuscript. AYL, BLG, CF, and BLP revised the manuscript. SCD, BLG, and BAR verified data analyses and interpretation. RYC and RB conducted the clinical study. JK provided clinical study space and coordinated the clinical study. CF and BLP conceived of the study and obtained funding.

## Conflict of Interest

The authors declare no conflicts of interest.
